# Trends in Resources for Neonatal Intensive Care at Delivery Hospitals for Infants Born Younger Than 30 Weeks’ Gestation, 2009-2020

**DOI:** 10.1001/jamanetworkopen.2023.12107

**Published:** 2023-05-05

**Authors:** Nansi S. Boghossian, Marco Geraci, Ciaran S. Phibbs, Scott A. Lorch, Erika M. Edwards, Jeffrey D. Horbar

**Affiliations:** 1 Department of Epidemiology and Biostatistics, Arnold School of Public Health, University of South Carolina, Columbia; 2 MEMOTEF Department, School of Economics, Sapienza University of Rome, Rome, Italy; 3 Health Economics Resource Center and Center for Implementation to Innovation, Veterans Affairs Palo Alto Health Care System, Menlo Park, California; 4 Perinatal Epidemiology and Health Outcomes Research Unit, Department of Pediatrics, Division of Neonatology, Stanford University School of Medicine, Stanford, California; 5 Division of Neonatology, Department of Pediatrics, The Children’s Hospital of Philadelphia, Perelman School of Medicine, University of Pennsylvania, Philadelphia; 6 Leonard Davis Institute of Health Economics, Wharton School, University of Pennsylvania, Philadelphia; 7 Vermont Oxford Network, Burlington, Vermont; 8 Department of Mathematics and Statistics, University of Vermont, Burlington; 9 Department of Pediatrics, University of Vermont College of Medicine, Burlington

## Abstract

**Importance:**

In an ideal regionalized system, all infants born very preterm would be delivered at a large tertiary hospital capable of providing all necessary care.

**Objective:**

To examine whether the distribution of extremely preterm births changed between 2009 and 2020 based on neonatal intensive care resources at the delivery hospital.

**Design, Setting, and Participants:**

This retrospective cohort study was conducted at 822 Vermont Oxford Network (VON) centers in the US between 2009 and 2020. Participants included infants born at 22 to 29 weeks’ gestation, delivered at or transferred to centers participating in the VON. Data were analyzed from February to December 2022.

**Exposures:**

Hospital of birth at 22 to 29 weeks’ gestation.

**Main Outcomes and Measures:**

Birthplace neonatal intensive care unit (NICU) level was classified as A, restriction on assisted ventilation or no surgery; B, major surgery; or C, cardiac surgery requiring bypass. Level B centers were further divided into low-volume (<50 inborn infants at 22 to 29 weeks’ gestation per year) and high-volume (≥50 inborn infants at 22 to 29 weeks’ gestation per year) centers. High-volume level B and level C centers were combined, resulting in 3 distinct NICU categories: level A, low-volume B, and high-volume B and C NICUs. The main outcome was the change in the percentage of births at hospitals with level A, low-volume B, and high-volume B or C NICUs overall and by US Census region.

**Results:**

A total of 357 181 infants (mean [SD] gestational age, 26.4 [2.1] weeks; 188 761 [52.9%] male) were included in the analysis. Across regions, the Pacific (20 239 births [38.3%]) had the lowest while the South Atlantic (48 348 births [62.7%]) had the highest percentage of births at a hospital with a high-volume B– or C-level NICU. Births at hospitals with A-level NICUs increased by 5.6% (95% CI, 4.3% to 7.0%), and births at low-volume B–level NICUs increased by 3.6% (95% CI, 2.1% to 5.0%), while births at hospitals with high-volume B– or C-level NICUs decreased by 9.2% (95% CI, −10.3% to −8.1%). By 2020, less than half of the births for infants at 22 to 29 weeks’ gestation occurred at hospitals with high-volume B– or C-level NICUs. Most US Census regions followed the nationwide trends; for example, births at hospitals with high-volume B– or C-level NICUs decreased by 10.9% [95% CI, −14.0% to −7.8%) in the East North Central region and by 21.1% (95% CI, −24.0% to −18.2%) in the West South Central region.

**Conclusions and Relevance:**

This retrospective cohort study identified concerning deregionalization trends in birthplace hospital level of care for infants born at 22 to 29 weeks’ gestation. These findings should serve to encourage policy makers to identify and enforce strategies to ensure that infants at the highest risk of adverse outcomes are born at the hospitals where they have the best chances to attain optimal outcomes.

## Key Points

**Question:**

Have neonatal intensive care resources at hospitals where infants born extremely preterm are delivered changed over the past decade?

**Findings:**

In this cohort study including 357 181 infants born at 22 to 29 weeks’ gestation between 2009 and 2020, births at neonatal intensive care units (NICUs) with lower levels of care or lower birth volumes increased, while births at NICUs with higher levels of care or higher birth volumes decreased.

**Meaning:**

The findings of this study suggest increasing deregionalization of extremely preterm birth.

## Teaser

This cohort study examines the distribution of extremely preterm births between 2009 and 2020 stratified by neonatal intensive care resources at the delivery hospital in the US.

## Introduction

In 1976, the March of Dimes Committee on Perinatal Health published recommendations on perinatal care regionalization in the US that included the referral of mothers and infants with high risk of adverse perinatal outcomes to a hospital with a regional neonatal intensive care unit (NICU).^1^ Regionalization was soon extensively implemented in the US, partly due to regulatory mechanisms organized by state certificate of need laws.^2^ Starting in the late 1980s and continuing into the 2000s, various studies, mostly from single states, found that perinatal deregionalization was occurring,^3,4,5,6,7,8,9,10,11^ with numbers of NICUs mainly increasing in urbanized areas that are within a reasonable distance to an existing tertiary NICU.^12^ However, to our knowledge, there are no recent data examining national perinatal regionalization trends.

A significant body of evidence demonstrates the importance of perinatal care regionalization, given the substantial reductions in mortality and morbidities for newborns at high risk of adverse outcomes, particularly infants with very low–birth weight (VLBW), delivered in hospitals with higher-level NICUs.^13,14,15,16,17^ An evolving body of literature also supports the importance of NICU volume, given the lower risk of mortality associated with being born at hospitals with higher-volume NICUs.^16,17,18,19,20,21,22,23,24,25^ However, to our knowledge, there are no data examining national trends in the distribution of infants with high risk in low- vs high-volume NICUs, nor have variations in these trends been examined by US region.

To address these issues, we used data from Vermont Oxford Network (VON), a nonprofit, voluntary, worldwide collaboration of health care professionals dedicated to improving the quality, safety, and value of care for newborns. We examined regionalization trends in the birthplace NICU level, incorporating NICU volume from 2009 to 2020 overall and by US region among newborns born at 22 to 29 weeks’ gestation.

## Methods

For this cohort study, the use of VON’s deidentified research repository was determined to be exempt from review and informed consent by the University of Vermont’s committee for human research because it was not human participants research. This study is reported following the Strengthening the Reporting of Observational Studies in Epidemiology (STROBE) reporting guideline.

### Study Sample

We included neonates born at 22 to 29 weeks’ gestation who were born at or transferred to VON US centers within 28 days of life from January 1, 2009, through December 31, 2020. eTable 1 in Supplement 1 shows the number of centers participating in VON throughout the study period.

### Study Variables

For VON members, NICU level is collected through the annual membership survey and classified as level A, restriction on assisted ventilation or no surgery; B, major surgery; or C, cardiac surgery requiring bypass. Given the strong association between the preterm infant admission volume and mortality,^16,17,18,19,20,21,22,23,24,25^ we further classified level B centers into low-volume (ie, <50 inborn infants at 22 to 29 weeks’ gestation per year) and high-volume (ie, ≥50 inborn infants at 22 to 29 weeks’ gestation per year). Since many studies have also found no outcome differences between high-volume B–level vs C-level centers (essentially all high volume),^18,20,25^ we combined high-volume B–level and C-level centers,^18,19,20,25^ resulting in 3 distinct NICU categories.

In January 2015, VON started collecting the names of the non-VON birth center for outborn infants transferring to a VON center. For these non-VON centers, we obtained the center’s level of neonatal care from publicly available resources. Level I centers were coded as having a well-baby nursery; level II, having an equivalent of VON’s classification of level A NICU; level III, having a level B NICU; and level IV, having a level C NICU. Centers with well-baby nursery and those with level A NICUs were combined into 1 group, labeled as level A NICUs. For outborn infants born before 2015, we did not know the NICU level of the non-VON center, so we only included infants transferred to VON centers within 3 days of life because we were more confident in assuming that these infants were being transferred from hospitals with level A NICUs or well-baby nurseries. NICU census regions and divisions were classified according to the US Census Bureau classifications as West (Mountain and Pacific), Midwest (East North Central and West North Central), South (South Atlantic, East South Central, and West South Central), and Northeast (New England and Mid-Atlantic) (eFigure 1 in Supplement 1).^26^ Member NICUs contribute data from medical records using standardized VON forms. Maternal race and ethnicity were obtained by personal interview with the mother, review of the birth certificate, or medical record, in that order. Race and ethnicity were classified as American Indian, Asian, Black, Hispanic, White, and other (for individuals who did not identify as any of the provided categories). Race and ethnicity were included in analysis as descriptive characteristics.

### Statistical Analysis

We conducted descriptive analyses examining the distribution of maternal and newborn characteristics by the birthplace level of neonatal care. We estimated national and regional trends in proportions of births in hospitals with A-level, low-volume B–level, or high-volume B– or C-level NICUs between 2009 and 2020. Models were run for all newborns at 22 to 29 weeks’ gestation and separately for newborns at 22 to 25 and 26 to 29 weeks’ gestation to assess any difference in trends between gestational age intervals. We used multinomial regression (nonproportional-odds cumulative logit model) to estimate the probability of birth in each NICU level, along with 95% CIs. We also calculated the absolute change in the percentage of births along with the 95% CI by NICU level between 2009 and 2020.

We conducted sensitivity analyses to determine the robustness of our findings. The first analysis was conducted restricting to hospitals that were in the sample the whole period. The second analysis recoded the missing NICU level into low-volume B instead of A for outborn infants transferred to VON centers within 3 days of life. A third analysis used a complete case analysis without recoding the missing level for outborn infants transferred within 3 days of life.

*P* values were 2-sided, and statistical significance was set at *P* = .05. Statistical analyses were conducted using R software version 4.0.5 (R Project for Statistical Computing). Data were analyzed from February to December 2022.

## Results

### Study Sample

From 2009 to 2020, 322 407 neonates were inborn at 822 VON centers and 39 652 were outborn and transferred to 714 VON centers. Among the inborn infants, 305 866 infants (94.9%) did not die in the delivery room and were admitted to the NICU. Among the outborn infants, 17 464 infants transferred from a birth center with a known NICU level. Among the remaining 22 188 outborn infants, 20 087 (90.5%) were transferred within 3 days of life (78.0% within 1 day, 11.8% within 2 days, and 0.8% within 3 days) and were classified as transferred from a hospital with a well-baby nursery or level A NICU, and 2100 (9.5%) were transferred after 3 days of life and were classified as missing birth NICU level (eFigure 2 in Supplement 1). The 2100 infants were subsequently transferred to VON centers with NICU levels A (4.8%), B (22.6%), or C (72.6%). For the final sample size, we excluded 2100 infants with missing data on birthplace level of newborn care, 2399 infants missing data on race and ethnicity, 337 infants missing data on congenital anomalies, and 124 with missing data on sex, resulting in 357 181 infants (mean [SD] gestational age, 26.4 [2.1] weeks; 188 761 [52.9%] male), including 320 243 inborn infants and 36 938 outborn infants. Overall, 108 283 infants received care at level A NICUs, 62 061 infants received care at low-volume level B NICUs, and 186 837 infants received care at high-volume level B or level C NICUs (Table 1).

**Table 1.** Maternal and Newborn Characteristics by Birth Hospital NICU Level in the Vermont Oxford Network

Characteristic	Infants by birth hospital NICU level, No. (%)^a^		
	A (n = 108 283)	Low-volume B (n = 62 061)	High-volume B or C (n = 186 837)
Inborn	74 957 (69.2)	58 940 (95.0)	186 346 (99.7)
**Region** ^b^			
New England	2565 (20.5)	2801 (22.4)	7140 (57.1)
Middle Atlantic	8592 (21.8)	10 150 (25.7)	20 699 (52.5)
East North Central	19 290 (34.4)	10 400 (18.6)	26 350 (47.0)
West North Central	5243 (20.4)	5990 (23.3)	14 439 (56.2)
South Atlantic	20 969 (27.2)	7823 (10.1)	48 348 (62.7)
East South Central	10 561 (44.0)	1253 (5.2)	12 184 (50.8)
West South Central	14 964 (30.0)	8686 (17.4)	26 273 (52.6)
Mountain	4457 (22.7)	4018 (20.5)	11 165 (56.9)
Pacific	21 642 (41.0)	10 940 (20.7)	20 239 (38.3)
**Volume, median (IQR)** ^c^			
New England	22 (15-65)	35 (25-40)	74 (61-95)
Middle Atlantic	37 (20-127)	34 (27-42)	69 (57-87)
East North Central	48 (28-101)	35 (27-42)	74 (62-97)
West North Central	24 (16-39)	32 (22-40)	94 (71-122)
South Atlantic	37 (25-62)	38 (30-45)	102 (76-127)
East South Central	39 (20-63)	38 (34-46)	110 (81-134)
West South Central	33 (18-50)	36 (25-43)	87 (62-130)
Mountain	25 (15-63)	29 (22-38)	78 (62-103)
Pacific	24 (15-34)	31 (21-40)	70 (58-94)
**Infant factors**			
Gestational age, mean (SD), wk	26.5 (2.0)	26.5 (2.0)	26.4 (2.1)
Congenital malformation	3962 (3.7)	2685 (4.3)	9801 (5.3)
Antenatal corticosteroids among inborn and outborn infants	76 907/107 589 (71.5)	52 274/61 905 (84.4)	162 930/186 508 (87.4)
Antenatal corticosteroids among inborn infants	61 511/74 803 (82.2)	49 890/58 842 (84.8)	162 525/186 023 (87.4)
Acute transfer among inborn and outborn infants^d^	52 918/103 958 (50.9)	8594/58 248 (14.8)	10 794/177 217 (6.1)
Acute transfer among inborn infants^e^	20 112/71 152 (28.3)	6604/56 258 (11.7)	10 362/176 785 (5.9)
Initial length of stay for inborn transferred infants, median (IQR), d^f^	16 (3-45)	40 (16-81)	51 (19-94)
Initial length of stay for inborn and outborn transferred infants, median (IQR), d^g^	1 (1-7)	23 (2-69)	47 (16-92)
Hospital ownership among inborn infants			
Government	9742 (13.0)	6499 (11.0)	21 086 (11.3)
Nonprofit	53 172 (71.1)	44 338 (75.3)	147 907 (79.4)
Investor	11 558 (15.4)	8046 (13.7)	16 316 (8.8)
Other	359 (0.5)	0 (0.0)	999 (0.5)
**Maternal factors**			
Race and ethnicity^h^			
American Indian and Alaska Native	846 (0.8)	591 (1.0)	1288 (0.7)
Asian	4288 (4.0)	3304 (5.3)	8114 (4.3)
Black	33 164 (30.6)	16 686 (26.9)	62 078 (33.2)
Hispanic	20 792 (19.2)	13 806 (22.3)	33 290 (17.8)
White	47 122 (43.5)	26 504 (42.7)	78 698 (42.1)
Other	2071 (1.9)	1170 (1.9)	3369 (1.8)
Chorioamnionitis^i^	4078 (14.7)	2717 (16.6)	7715 (18.8)
Hypertension^i^	7241 (25.9)	5046 (30.7)	13 797 (33.7)
Multiple gestation	23 543 (21.7)	14 928 (24.0)	48 555 (26.0)

Abbreviation: NICU, neonatal intensive care unit.

^a^ Data were missing for 1179 inborn and outborn infants on antenatal corticosteroids, including 575 inborn infants; 221 infants on hospital ownership; 711 mothers on chorioamnionitis; 439 mothers on hypertension; 12 mothers on multiple gestation. Infants born at non-VON centers and transferred prior to 3 days of life coded as being born at a hospital with well-baby nursery/NICU level A.

^b^ Row percentages reported.

^c^ Volume variable restricted to VON centers and includes inborn infants only.

^d^ Acute transfer excludes delivery room deaths and includes both inborn infants and outborn infants who were transferred to VON centers within 3 days of life. Acute transfer defined as transfer for medical or diagnostic services or surgery for infants born at VON centers and as transfer within 3 days of life for outborn infants.

^e^ Acute transfer excludes delivery room deaths. Acute transfer defined as transfer for medical or diagnostic services or surgery for infants born at VON centers.

^f^ Data were available for 20 090 infants at level A NICUs, 6602 infants at low-volume B-level NICUs, and 10 361 infants at high-volume B– or C-level NICUs.

^g^ Includes infants transferred from VON and non-VON centers. Data were available for 52 895 infants at level A NICUs, 8592 infants at low-volume B-level NICUs, and 10 793 infants at high-volume B– or C-level NICUS.

^h^ Maternal race and ethnicity were obtained by personal interview with the mother, review of the birth certificate, or medical record, in that order. The other category indicates that none of the race categories applied to the biological mother.

^i^ Data collection on hypertension and chorioamnionitis variables started in 2008.

Among 74 957 inborn infants delivered at hospitals with level A NICUs, 7164 (9.6%) were born at centers with restricted ventilation. Table 1 shows maternal and newborn characteristics by birth hospital NICU level. Across regions, the Pacific (20 239 infants [38.3%]) had the lowest while the South Atlantic (48 348 infants [62.7%]) had the highest percentage of births at a hospital with a high-volume B– or C-level NICU. Among VON centers with level A NICUs, the median (IQR) volume of infants born at 22 to 29 weeks’ gestation ranged between 22 (15-65) infants per year in New England and 48 (28-101) infants per year in the East North Central region, while among high-volume B– and C-level VON NICUs, it ranged between 70 (58-94) infants per year in the Pacific and 110 (81-134) infants per year in the East South Central region. Infants with congenital anomalies were more likely to be born at hospitals with high-volume B– or C-level NICUs (9801 infants [5.3%]) and less likely to be born at hospitals with level A NICUs (3962 infants [3.7%]). Acute transfer among inborn infants was higher if newborns were delivered at hospitals with level A NICUs (20 112 of 71 152 infants [28.3%]) and lower if newborns were delivered at hospitals with high-volume B– or C-level NICUs (10 362 of 176 785 infants [5.9%]). Hospitals with level A and low-volume B–level NICUs were more likely to be investor owned and less likely to be nonprofit owned, while hospitals with high-volume B– or C-level NICUs were less likely to be investor owned and more likely to be nonprofit owned (Table 1). Individuals with pregnancy complications and multiple gestations were more likely to deliver at hospitals with high-volume B– or C-level NICUs and less likely to deliver at hospitals with level A NICUs (Table 1).

### National Trends Between 2009 and 2020

**Figure 1.**
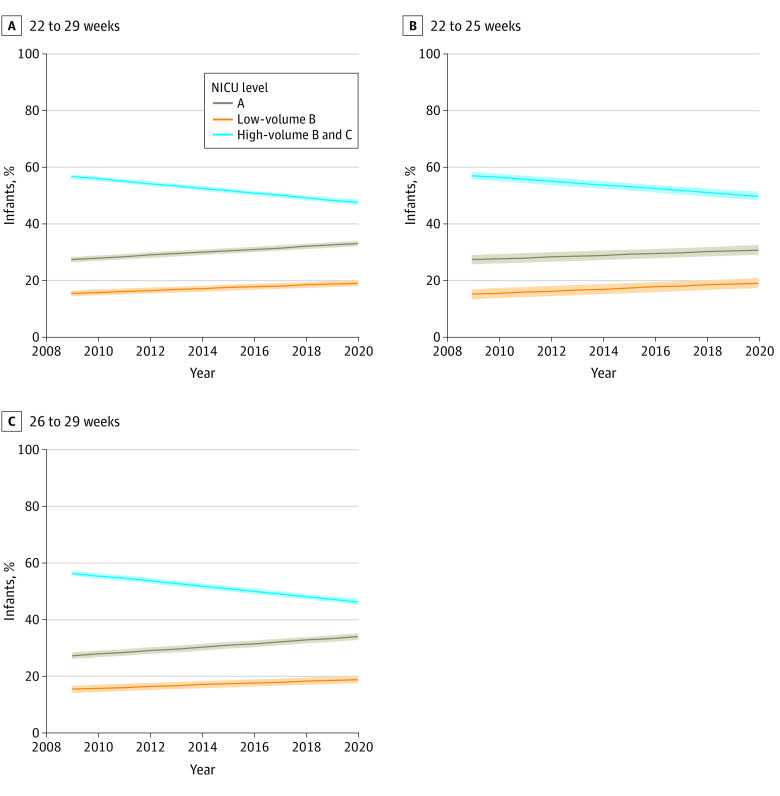
National Trend of Births by NICU Level Among Newborns Born at 22 to 29 Weeks’ Gestation Solid lines represent percentages; shading, 95% CIs.

Figure 1 shows the national trend of births in A-, low-volume B–, and high-volume B– or C-level NICUs among newborns born at 22 to 29 weeks’ gestation and for the 22- to 25-week and 26- to 29-week subsets of these infants. Between 2009 and 2020, births at 22 to 29 weeks’ gestation at level A NICUs increased by 5.6% (95% CI, 4.3% to 7.0%) and births at and low-volume B–level NICUs increased by 3.6% (95% CI, 2.1% to 5.0%), while births at high-volume B– or C-level NICUs decreased by 9.2% (95% CI, −10.3% to −8.1%) (Table 2). By 2020, fewer than half of newborns born at less than 30 weeks’ gestation were born in high-volume B– or C-level centers. These trends were also observed among infants born at 22 to 25 and 26 to –29 weeks gestation infants, with the shift away from high-volume B– or C-level centers being more pronounced among infants born at 26 to 29 weeks gestation (Table 2).

**Table 2.** Percentage of Births by NICU Level in 2009 and 2020 Among Newborns Born at 22 to 29 Weeks’ Gestation

Gestational age, wk	Level A NICU^a^			Low-volume B–level NICU			High-volume B– or C- level NICU, % (95% CI)		
	% (95% CI)		Absolute change, percentage points (95% CI)	% (95% CI)		Absolute change, percentage points (95% CI)	% (95% CI)		Absolute change, percentage points (95% CI)
	2009	2020		2009	2020		2009	2020	
22-29	27.6 (26.6 to 28.5)	33.2 (32.2 to 34.2)	5.6 (4.3 to 7.0)	15.6 (14.6 to 16.6)	19.1 (18.1 to 20.2)	3.6 (2.1 to 5.0)	56.8 (56.1 to 57.6)	47.6 (46.8 to 48.5)	−9.2 (−10.3 to −8.1)
22-25	27.5 (25.8 to 29.2)	30.9 (29.2 to 32.6)	3.4 (1.0 to 5.8)	15.3 (13.6 to 17.1)	19.2 (17.4 to 21.0)	3.9 (1.3 to 6.4)	57.2 (55.9 to 58.4)	49.9 (48.4 to 51.4)	−7.3 (−9.2 to −5.3)
26-29	27.6 (26.4 to 28.8)	34.3 (33.2 to 35.5)	6.7 (5.1 to 8.4)	15.7 (14.5 to 17.0)	19.1 (17.8 to 20.4)	3.4 (1.6 to 5.2)	56.7 (55.8 to 57.6)	46.5 (45.5 to 47.6)	−10.1 (−11.5 to −8.7)

Abbreviation: NICU, neonatal intensive care unit.

^a^ For outborn newborns transferred within 3 days of life to VON centers, NICU level coded as level A.

### Regional Trends Between 2009 and 2020

**Figure 2.**
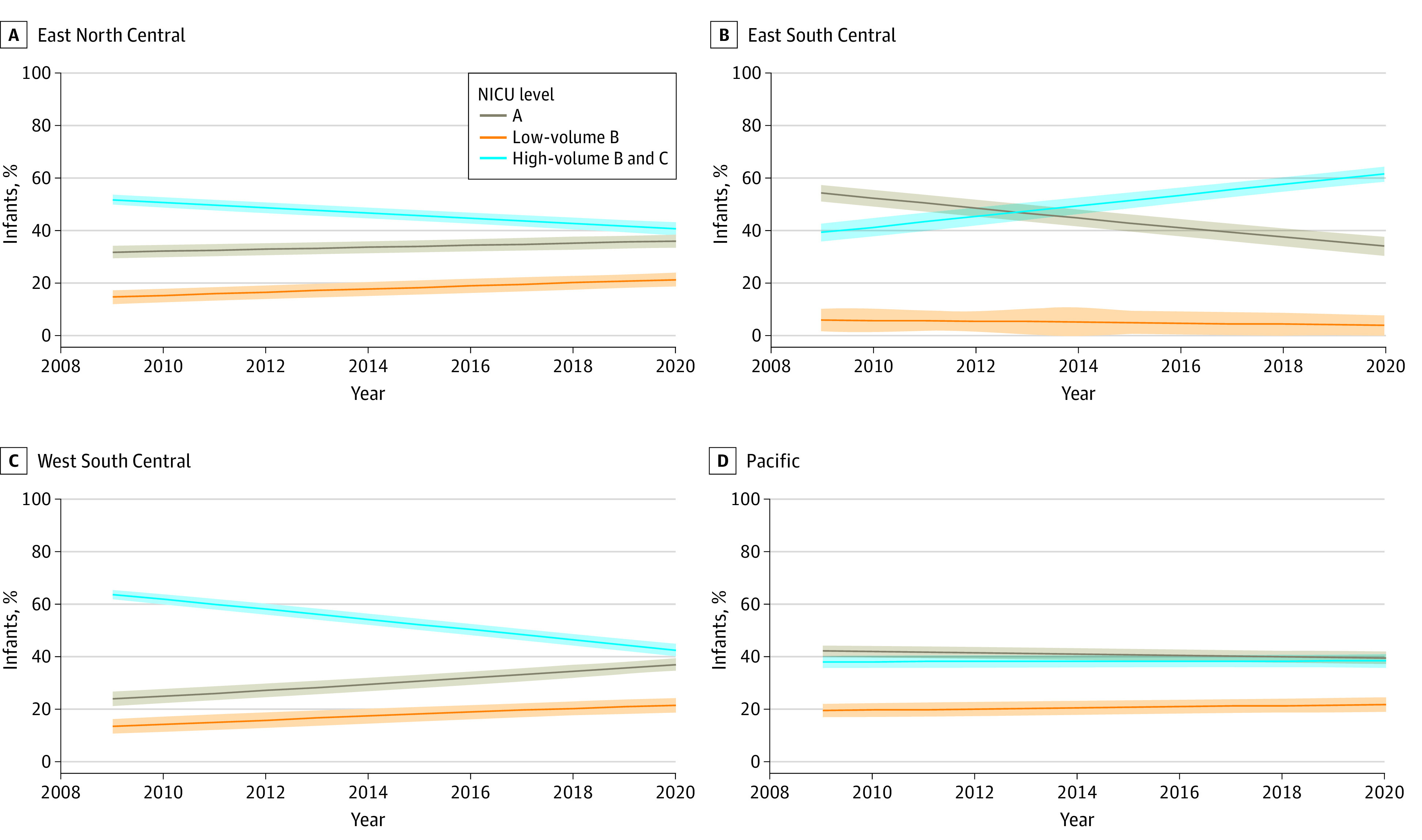
Regional Trend of Births by NICU Level Between 2009 and 2020 Among 22-to-29–Week Newborns Only 4 regions are displayed. The complete data for all regions are presented in eFigure 3 in Supplement 1. Solid lines represent percentages; shading, 95% CIs.

Among infants born at 22 to 29 weeks’ gestation, most regions followed the nationwide trends (eFigure 3 in Supplement 1). The exceptions were the Pacific, which started with a much smaller share of these infants at high-volume B– or C-level centers and showed some small changes in the site of delivery during the study period, and the East South Central region, which had a lower regionalization rate at the start of the study and shifted toward the national mean by the end of the study (Table 3 and Figure 2). Between 2009 and 2020, the largest declines were observed in New England (−21.2%; 95% CI, −27.1% to −15.3%) and the West South Central region (−21.1%; 95% CI, −24.0% to −18.2%), while the East South Central region had the largest increase (22.2%; 95% CI, 17.8% to 26.6%) in births at high-volume B– or C-level NICUs (Table 3). In 2020, the Pacific had the lowest number of births at high-volume B– or C-level NICUs (38.5%; 95% CI, 35.9% to 41.0%). The West South Central region had the largest increase (8.1%; 95% CI, 4.2% to 11.9%), while the West North Central region had the largest decrease (−8.0%; 95% CI, −13.3% to −2.7%) in births at low-volume B–level NICUs. New England had the largest increase (14.0%; 95% CI, 6.2% to 21.9%) while the East South Central region had the largest decrease (−20.2%; 95% CI, −24.9% to −15.4%) in the percentage of births at level A NICUs.

**Table 3.** Percentage of Births by NICU Level and Region in 2009 and 2020 Among Newborns Born at 22 to 29 Weeks’ Gestation

Region	Level A NICU^a^			Low-volume B–level NICU			High-volume B– or C-level NICU		
	% (95% CI)		Absolute change, percentage points (95% CI)	% (95% CI)		Absolute change, percentage points (95% CI)	% (95% CI)		Absolute change, percentage points (95% CI)
	2009	2020		2009	2020		2009	2020	
New England	14.1 (8.7 to 19.5)	28.2 (22.5 to 33.9)	14.0 (6.2 to 21.9)	18.5 (13.1 to 23.9)	25.7 (20.4 to 30.9)	7.1 (−0.4 to 14.7)	67.3 (63.9 to 70.8)	46.2 (41.3 to 51.0)	−21.2 (−27.1 to −15.3)
Middle Atlantic	18.5 (15.2 to 21.8)	25.5 (22.7 to 28.3)	7.0 (2.7 to 11.4)	22.6 (19.6 to 25.6)	28.8 (25.8 to 31.9)	6.2 (1.9 to 10.5)	58.9 (56.9 to 60.9)	45.7 (42.8 to 48.5)	−13.3 (−16.8 to −9.8)
East North Central	32.4 (30.0 to 34.8)	36.6 (34.2 to 39.1)	4.2 (0.8 to 7.6)	15.3 (12.8 to 17.8)	22.0 (19.4 to 24.6)	6.7 (3.1 to 10.3)	52.3 (50.4 to 54.2)	41.4 (38.9 to 43.9)	−10.9 (−14.0 to −7.8)
West North Central	15.0 (10.8 to 19.2)	26.7 (22.5 to 30.8)	11.7 (5.8 to 17.6)	26.9 (23.3 to 30.6)	18.9 (15.1 to 22.8)	−8.0 (−13.3 to −2.7)	58.1 (55.4 to 60.7)	54.4 (51.5 to 57.3)	−3.7 (−7.6 to 0.3)
South Atlantic	22.1 (19.9 to 24.3)	32.7 (30.6 to 34.8)	10.6 (7.5 to 13.6)	7.5 (5.3 to 9.7)	12.8 (10.4 to 15.2)	5.3 (2.1 to 8.6)	70.4 (69.1 to 71.7)	54.5 (52.8 to 56.2)	−15.9 (−18.1 to −13.8)
East South Central	54.4 (51.3 to 57.5)	34.3 (30.7 to 37.8)	−20.2 (−24.9 to −15.4)	6.1 (1.9 to 10.3)	4.1 (0.3 to 7.9)	−2.0 (−7.7 to 3.7)	39.5 (36.1 to 42.8)	61.6 (58.8 to 64.5)	22.2 (17.8 to 26.6)
West South Central	23.6 (20.9 to 26.3)	36.6 (34.2 to 39.0)	13.0 (9.4 to 16.6)	13.1 (10.3 to 15.9)	21.2 (18.5 to 23.9)	8.1 (4.2 to 11.9)	63.3 (61.4 to 65.1)	42.2 (39.9 to 44.5)	−21.1 (−24.0 to −18.2)
Mountain	18.0 (13.5 to 22.6)	27.9 (23.6 to 32.1)	9.8 (3.6 to 16.1)	20.9 (16.5 to 25.2)	19.7 (15.5 to 23.8)	−1.2 (−7.2 to 4.8)	61.1 (58.0 to 64.2)	52.5 (48.8 to 56.2)	−8.6 (−13.4 to −3.8)
Pacific	42.2 (40.1 to 44.4)	39.6 (37.2 to 42.0)	−2.6 (−5.8 to 0.6)	19.6 (17.2 to 22.0)	21.9 (19.1 to 24.7)	2.3 (−1.4 to 6.0)	38.2 (35.9 to 40.4)	38.5 (35.9 to 41.0)	0.3 (−3.1 to 3.7)
All	27.6 (26.6 to 28.5)	33.2 (32.2 to 34.2)	5.6 (4.3 to 7.0)	15.6 (14.6 to 16.6)	19.1 (18.1 to 20.2)	3.6 (2.1 to 5.0)	56.8 (56.1 to 57.6)	47.6 (46.8 to 48.5)	−9.2 (−10.3 to −8.1)

Abbreviation: NICU, neonatal intensive care unit.

^a^ For outborn newborns transferred within 3 days of life to VON centers, NICU level coded as level A.

When restricting to infants born at 22 to 25 weeks’ gestation, results were similar to the cohort of infants born at 22 to 29 weeks’ gestation (eFigure 4 in Supplement 1). Between 2009 and 2020, decreasing trends of births at high-volume B– or C-level NICUs occurred across all regions except the East South Central region and Pacific (eTable 2 in Supplement 1). However, by 2020, the Pacific region had the lowest number of births at high-volume B– or C-level NICUs (41.8%; 95% CI, 37.3% to 46.4%). The Middle Atlantic and West South Central regions had the largest increases while the West North Central region had the largest decrease in births at low-volume B–level NICUs. By 2020, the Middle Atlantic had the highest percentage of births at low-volume B–level NICUs (30.7%; 95% CI, 25.1% to 36.3%). New England had the largest increase while the East South Central region had the largest decrease in the percentage of births at level A NICUs (eTable 2 in Supplement 1).

Between 2009 and 2020, decreasing trends of births at high-volume B– or C-level NICUs for infants born at 26 to 29 weeks’ gestation occurred across all regions except the East South Central region (eFigure 5 in Supplement 1). Between 2009 and 2020, increasing trends of births at low-volume B–level NICUs occurred across all regions except the West North Central region, East South Central, and Mountain regions (eTable 3 in Supplement 1). By 2020, the Middle Atlantic had the highest percentage of births at low-volume B–level NICUs (28.0%; 95% CI, 24.3% to 31.6%). New England and the West South Central region had the largest increase while the East South Central region had the largest decrease in the percentage of births at level A NICUs.

### Sensitivity Analyses

Restricting to hospitals that were in the sample the whole period showed similar national trends, although the changes between 2009 and 2020 were smaller (eTable 4 in Supplement 1). The changes comparing the whole to the restricted sample were less pronounced if the region did not experience the addition of several new hospitals (eTable 5 in Supplement 1). Between 2009 and 2020 among newborns at 22 to 29 weeks’ gestation, births decreased at hospitals with high-volume B– or C-level NICUs, while births increased at hospitals with level A NICUs. Depending on the sensitivity analysis, births at low-volume B–level NICUs either decreased or increased (eTable 6 and eTable 7 in Supplement 1).

## Discussion

In this cohort study using a national sample, we found a nationwide shift toward deregionalization of perinatal care among infants born at 22 to 29 weeks’ gestation. Between 2009 and 2020, births across all regions increased at level A by 5.6% and at low-volume B–level NICUs by 3.6%, while births at high-volume B– or C-level NICUs decreased by 9.2%. By 2020, fewer than half of these births occurred at hospitals with high-volume B– or C-level NICUs, settings associated with significantly lower rates of mortality and serious morbidity.^13,14,15,16,17,18,19,20,21,22,23,24,25,27^ Similar trends were observed among infants born at 22 to 25 and 26 to 29 weeks’ gestation, with the shift away from the high-volume B– or C-level centers being more pronounced among infants born at 26 to 29 weeks’ gestation.

Previous research has shown the importance of birth at a hospital with a high-level NICU for infants who are very preterm or have VLBW.^13,14,15,16,17^ In a meta-analysis of studies published through 2010, birth in a nonregional hospital, compared with a regional hospital, was associated with increased odds of mortality (adjusted odds ratio [aOR], 1.55; 95% CI, 1.21-1.98) among infants born at 32 weeks’ gestation or less.^13^ This association was even stronger among infants weighing less than 1000 g (aOR, 1.80; 95% CI, 1.31-2.46).^13^ Another study showed that among newborns younger than 28 weeks’ gestation in a tertiary hospital, birth in a nontertiary hospital was associated with increased odds of mortality (OR, 2.32; 95% CI, 1.78-3.06), while transfer from a nontertiary to a tertiary hospital in the first 48 hours was associated with increased odds of severe brain injury.^27^ In addition to the large number of studies on the benefits associated with being born at a hospital with a high-level NICU, an evolving body of evidence emphasizes the importance of NICU volume.^16,17,18,19,20,21,22,23,24,25^ Collectively, these studies provide strong evidence that the best outcomes are associated with delivery at high-level and high-volume NICUs.^13,14,15,16,17,18,19,20,21,22,23,24,25,27^

However, previous studies have underestimated the benefits associated with regionalization and with NICU level and NICU volume. Given that patients who are more severely ill are more likely to deliver at hospitals with high-level and high-volume NICUs, studies conducted using instrumental variables methods that control for this unobserved selection bias show even stronger associations with improved neonatal outcomes when infants with high risk of adverse outcomes are delivered at high-level and high-volume NICUs, compared with low-level and low-volume NICUs.^14,22,23^ For example, a study by Wehby et al^23^ found that being delivered at hospitals with low-volume NICUs (<50 infants with VLBW per year), compared with high-volume NICUs (>100 infants with VLBW per year), was associated with nearly 2-fold (aOR, 1.80; 95% CI, 1.09-2.99) increased mortality odds under the classic risk-adjusted model, compared with more than 5-fold (aOR, 5.43; 95% CI, 1.21-27.47) increased mortality odds using the instrumental variable approach.^23^

We speculate that the increasing share of infants delivered in hospitals with level A or low-volume B–level NICUs is accounted for by a decrease in the share of infants delivered in hospitals with high-volume B– or C-level NICUs. A study conducted in California examined how delivery location shifted in response to midlevel NICU openings (equivalent to our level A or low-volume B–level).^6^ Almost all (88%) of the VLBW deliveries at these new NICUs were shifted from hospitals with a high-volume B– or C-level NICU.^6^ Thus, this increase in midlevel NICUs was essentially all deregionalization, not improved NICU care access.^6^

The proliferation of midlevel NICUs is mainly happening in urban and suburban areas that are already being served by tertiary centers.^6,7^ In California, for example, approximately 80% of births that occurred in smaller, lower-level NICUs were located within 25 miles of a large tertiary NICU.^12^ This deregionalization in perinatal care has occurred due to the wide availability of neonatal intensive care technologies and neonatologists, economic factors and financial incentives derived from installing new NICUs,^12,28^ and different state policies that influence regulation of regionalized systems.^2,29^ The effects of these different factors have also varied by US region, contributing to the regionalization trends we observed.

Between 2009 and 2020, births at high-volume B– or C-level NICUs decreased between 3.7% and 21.2% and births at level A NICUs increased between 4.2% and 14.0%. Births at low-volume B–level NICUs increased across 6 regions, and the increase ranged between 2.3% and 8.1%. Only 2 regions stood out in their regionalization trends: the East South Central and Pacific regions. The East South Central region had a lower regionalization rate at the start of the study and shifted toward the national mean by the end, while the Pacific region had a minor change in the delivery site during the study period because this region had already experienced a decline in the level of NICU care regionalization.

Most births at 22 to 29 weeks’ gestation (75%)^30^ in the Pacific region occur in California. California had already shown deregionalization trends starting in the 1990s.^5,20,31^ This trend continued and between 1990 and 2001: there were 48 new midlevel units established or upgraded,^6^ and between 2005 and 2011, the overall percentage of infants with VLBW born at hospitals providing the highest degree of care (defined as centers providing major surgery with >100 infants with VLBW per year and centers providing major surgery, extracorporeal membrane oxygenation, and cardiopulmonary bypass) decreased from 42.5% to 26.5%.^19^

Given the negative outcomes associated with birth at nontertiary or low-volume hospitals, the fact that more than 50% of these infants with high risk were born in hospitals with level A and low-volume B–level NICUs is unacceptable. Previous studies have shown that the delivery of these infants with high risk can be shifted to tertiary centers if there is a systemwide effort. In 1990, Portugal closed all the small maternity units and small NICUs and implemented a system to facilitate maternal transport of high-risk deliveries.^32^ This resulted in more than 90% of all very preterm deliveries occurring in a hospital with a high-volume NICU, which was associated with decreasing the neonatal mortality rate from 8.1 deaths to 2.7 deaths per 1000 live births.^32^ On a local level, in the greater Cincinnati, Ohio, region, the implementation of perinatal outreach programs stressing the importance of transfer of mothers with high risk to subspecialty perinatal centers decreased the percentage of infants with VLBW delivered at hospitals without tertiary perinatal care from 25% to 11.8%.^33^

### Limitations

This study has several limitations. One limitation is the missing data on the birth hospital level for a large proportion of outborn infants. We analyzed our data by region, which might mask individual state-level differences. To classify the NICU level for outborn infants, we used publicly available resources, which might have incomplete and inaccurate web descriptions that claim a higher level of care.^34^

## Conclusions

In this cohort study of neonates born at 22 to 29 weeks’ gestation, we identified concerning deregionalization trends. Given the strong evidence showing risk of worse outcomes when the birth of newborns with high risk does not occur at large tertiary centers, our findings should serve to encourage policy makers to identify and enforce strategies that ensure that infants with the highest risk of adverse outcomes are born at the hospitals where they have the best chance to attain optimal outcomes.

## Notes

Supplement 1. **eTable 1.** US Vermont Oxford Network Participating Centers by Region Between 2009 and 2020 **eFigure 1.** US Census Regions and Divisions **eFigure 2.** Disposition Status of Inborn and Outborn Infants Born at 22-29 Weeks’ Gestation Between 2009 and 2020 in the Vermont Oxford Network **eFigure 3.** Regional Trend of Births by NICU Level Between 2009 and 2020 Among Newborns Born at 22-29 Weeks’ Gestation **eFigure 4.** Regional Trend of Births by NICU Level Between 2009 and 2020 Among Newborns Born at 22-25 Weeks’ Gestation **eTable 2.** Percentage of Births by NICU Level by Region in 2009 and 2020 Restricting to Newborns Born at 22-25 Weeks’ Gestation **eFigure 5.** Regional Trend of Births by NICU Level Between 2009 and 2020 Among Newborns Born at 26-29 Weeks’ Gestation **eTable 3.** Percentage of Births by NICU Level by Region in 2009 and 2020 Restricting to Newborns Born at 26-29 Weeks’ Gestation **eTable 4.** Percentage of Births by NICU Level in 2009 and 2020 Among Preterm Newborns Restricting to Centers Participating in VON Throughout the Whole Study Period **eTable 5.** Percentage of Births by NICU Level and Region in 2009 and 2020 Among Newborns Born at 22-29 Weeks’ Gestation Restricting to Centers Participating in VON Throughout the Whole Study Period **eTable 6.** Sensitivity Analysis: Percentage of Births by NICU Level in 2009 and 2020 Among Newborns Born at 22-29 Weeks’ Gestation **eTable 7.** Sensitivity Analysis Restricted to Newborns With Nonmissing Birth NICU Level Data: Percentage of Births by NICU Level in 2009 and 2020 Among Newborns Born at 22-29 Weeks’ Gestation **eTable 8.** Vermont Oxford Network Participating Centers Between 2009 and 2020

Supplement 2.
Data Sharing Statement
