# Supplementary material for: Trends in Resources for Neonatal Intensive Care at Delivery Hospitals for Infants Born Younger Than 30 Weeks’ Gestation, 2009-2020
Source: JAMA Netw Open. 2023 May 5;6(5):e2312107. doi: 10.1001/jamanetworkopen.2023.12107 (PMC10163386; doi:10.1001/jamanetworkopen.2023.12107)
Supplement: Supplement 2. — Data Sharing Statement [file jamanetwopen-e2312107-s002.pdf]

Data Sharing Statement

Boghossian. Trends in Resources for Neonatal Intensive Care at Delivery Hospitals for Infants Born Younger Than 30 Weeks' Gestation, 2009-2020. *JAMA Netw Open*. Published May 05, 2023. doi:10.1001/jamanetworkopen.2023.12107

Data
Data available: No
